# Vitamin A and Its Related Diseases

**DOI:** 10.1002/fsn3.70630

**Published:** 2025-07-15

**Authors:** Shuhan Rao, Tiewei Li, Xiaojuan Li, Ligong Hou, Wan Sun

**Affiliations:** ^1^ Department of Clinical Laboratory, Zhengzhou Key Laboratory of Children's Infection and Immunity Children's Hospital Affiliated to Zhengzhou University Zhengzhou China; ^2^ Henan International Joint Laboratory of Prevention and Treatment of Pediatric Diseases Children's Hospital Affiliated to Zhengzhou University Zhengzhou China

**Keywords:** autoimmune diseases, eye diseases, infectious diseases, metabolic diseases, vitamin A

## Abstract

Vitamin A (VA), an essential micronutrient, plays a pivotal role in human growth, development, immune function, and vision. Vitamin A deficiency not only affects children's vision and growth, but also increases their susceptibility to infectious diseases, such as respiratory and gastrointestinal infections. Deficiency in vitamin A not only disrupts growth and development in children but also increases susceptibility to infectious diseases, such as respiratory and gastrointestinal infections. From an immunological standpoint, vitamin A is essential for the proper development and function of the immune system, with its deficiency impairing immune responses. Emerging evidence also suggests a role for vitamin A in the pathogenesis of systemic diseases, including metabolic and autoimmune disorders. Therefore, monitoring and managing vitamin A levels are of great significance in disease prevention and treatment. To fully understand the mechanism of vitamin A in various clinical diseases, it is important to provide a theoretical basis for improving the nutritional status of vitamin A to reduce the incidence of diseases and improve patient outcomes.

## Graphical abstract

Vitamin, an essential micronutrient, is pivotal in human growth, development, immune function, and vision. Deficiency in VA disrupts growth and development in children and increases susceptibility to infectious diseases, such as respiratory and gastrointestinal infections. A comprehensive understanding of VA's involvement in clinical diseases is vital for effective disease prevention and treatment, providing a theoretical basis for interventions to improve VA nutritional status, thereby reducing disease incidence and enhancing patient outcomes.

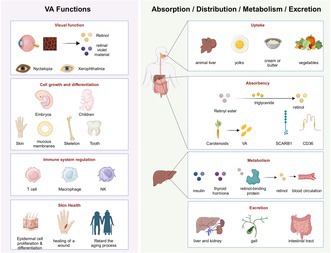

## 1 Introduction

Vitamin A, a vital yet frequently deficient fat‐soluble vitamin, is essential for normal metabolism and various physiological functions (Zhang, Dai, et al. 2023; Zhu 2010). This terpenoid compound, with the molecular formula C2OH3OO, exists in several forms, including retinol, retinaldehyde, retinoic acid (RA), retinyl acetate, and retinyl palmitate, with retinol, retinaldehyde, and RA being the most prevalent (Zhu 2010). Retinol, primarily stored in the liver, is present in both animal and plant tissues, while retinal, a precursor to rhodopsin in the retina, plays a critical role in visual health (Blaner et al. 2016; Carazo et al. 2021). RA, one of vitamin A's active forms, is essential for regulating cell growth, differentiation, and immune function (Szymański et al. 2020). Beyond its role in vision, vitamin A is fundamental to immune regulation, cell differentiation, and the growth and development of numerous physiological processes (Sajovic et al. 2022; Abdelkader et al. 2022).

With the decline in the mortality rate of infectious diseases, immune‐mediated chronic diseases have become the main burden of public health problems, and vitamin A happens to be the core nutrient for immune regulation. According to data from the United Nations Children's Fund, 18% of children under the age of 6 in Turkey suffer from subclinical vitamin A deficiency, and 2600 children die each year as a result (UNICEF 2004). According to the 2022 Turkish Nutrition and Health Survey data, 26.6% of people over the age of 15 have insufficient vitamin A intake (Tosyali et al. 2025). A survey on the serum vitamin A levels of children in China found that the vitamin A deficiency rate among children in Zhoushan City, Jiangsu Province, was 16.96% in 2021, and the vitamin A deficiency rate among children aged 0–3 in Hainan Province was 9.33% in 2024 (Yao et al. 2021; Zhao et al. 2024). Although the economic development of contemporary society and the upgrading of the logistics system have significantly improved the accessibility of vitamin A‐rich foods, the lack of health literacy among parents and the inertial effect of traditional dietary patterns still constitute the key social determinants affecting children's vitamin A nutritional status. The marginal deficiency rate of vitamin A among children in our country remains at a relatively high level, which is similar to the situation in most emerging developing countries (Stevens et al. 2015; Pfeiffer et al. 2013). Vitamin A deficiency has a significant impact on children's growth and development, potentially leading to vision loss and an increased risk of infections (Xu et al. 2021; Aibana et al. 2017). Moreover, vitamin A deficiency is a potential risk factor for cognitive impairment and mental disorders (Liu et al. 2016; Wołoszynowska‐Fraser et al. 2020). Therefore, it is regarded as the second‐largest risk factor for global disease burden (Zhao et al. 2022).

Given the significant importance of vitamin A in public health, the disease burden caused by vitamin A deficiency urgently needs attention. Therefore, recent studies have increasingly focused on the correlation between vitamin A and clinical diseases. This article reviews the latest research progress on vitamin A in ocular diseases, respiratory system diseases, intestinal diseases, infectious diseases, endocrine diseases, and autoimmune diseases, providing references for clinical treatment and guiding the direction of subsequent research.

### 1.1 Absorption, Distribution, Metabolism, and Elimination of Vitamin A

Dietary sources of vitamin A include animal‐based foods such as liver, egg yolks, and cream, as well as plant‐based foods like carrots, spinach, pumpkins, and kale (Gao et al. 2024; Debelo et al. 2017). In animal‐derived products such as liver, egg yolk, and cream, vitamin A exists in the form of retinol and its cis‐trans isomers, which are metabolized to retinol in the gastrointestinal lumen by enzymes like triglyceride lipase or phospholipase B before entering enterocytes. Retinol uptake occurs via either active transport or passive diffusion (Gao et al. 2024; Quick and Ong 1990). Once inside enterocytes, retinol binds to cellular retinol‐binding proteins, is then converted into retinyl esters, and incorporated into chylomicrons, which enter the circulation via the lymphatic system (Carazo et al. 2021; Choi et al. 2021; Perusek and Maeda 2013; Prakash 2006; Zhong et al. 2012; Ong and Chytil 1978). In contrast, plant‐based foods provide β‐carotene, which is poorly absorbed in the intestine. β‐carotene absorption occurs through transporters such as scavenger receptor B1 (SCARB1) and cluster of differentiation 36 (CD36) (Carazo et al. 2021). Some carotenoids are converted into active vitamin A forms in intestinal cells, with conversion regulated by various factors (Carazo et al. 2021). Unmetabolized carotenoids may enter the circulation or be stored in adipose tissue (Carazo et al. 2021).

The liver serves as the main storage site for vitamin A, where it is stored as retinyl ester in lipid droplets, ensuring stability and preventing cytotoxicity (Sajovic et al. 2022; Futterman and Andrews 1964). Upon entering the liver, some retinyl ester is hydrolyzed to retinol, which binds to retinol‐binding protein (RBP) (Futang 2015; Kam et al. 2012; Newcomer and Ong 2000; Soprano et al. 1986). This complex enables its secretion into the bloodstream and subsequent distribution to target tissues (Kanai et al. 1968). In the bloodstream, retinol binds to transthyretin (TTR), forming a stable complex that is essential for the proper delivery of retinol to target cells and prevents RBP degradation in the kidney (Raz and Goodman 1969; Goodman 1984; Peterson and Berggård 1971; Episkopou et al. 1993).

Vitamin A metabolism is a complex process involving multiple enzymes, proteins, and metabolic pathways, primarily occurring in the liver and intestinal epithelial cells (Xiao 2002). This process is tightly regulated, with hormones such as insulin and thyroid hormone playing significant roles in the storage and release of vitamin A. Additionally, metabolic pathways are subject to negative feedback regulation by vitamin A and its metabolites to ensure homeostasis and prevent toxicity (O'Connor et al. 2022). In the liver, intracellular retinol is oxidized to retinaldehyde under the catalysis of alcohol dehydrogenase (ADH) or retinol dehydrogenase (RDH, such as RDH10); retinaldehyde is further irreversibly converted to all‐trans retinoic acid (ATRA) under the action of aldehyde dehydrogenase (ALDH, such as ALDH1A1, ALDH1A2) (von Lintig 2012; Defnet et al. 2021). RA, a key active form of vitamin A, regulates gene expression by binding to intracellular retinoic acid‐binding proteins (CRABP) and nuclear receptors, influencing cell proliferation, differentiation, and function (Carazo et al. 2021). RA is mainly metabolized by the cytochrome P450 enzyme system, producing more polar metabolites that are involved in cell growth, differentiation, and apoptosis (Carazo et al. 2021). Most vitamin A metabolites are transported from the liver to the intestine via bile; some are excreted in the feces, and the remainder is reabsorbed and recirculated through the enterohepatic circulation (Barua and Olson 1986). Specific vitamin A metabolites are also excreted in the urine. Under normal conditions, vitamin A is excreted slowly to maintain stable levels in the body.

### 1.2 Function of Vitamin A

**FIGURE 1:**
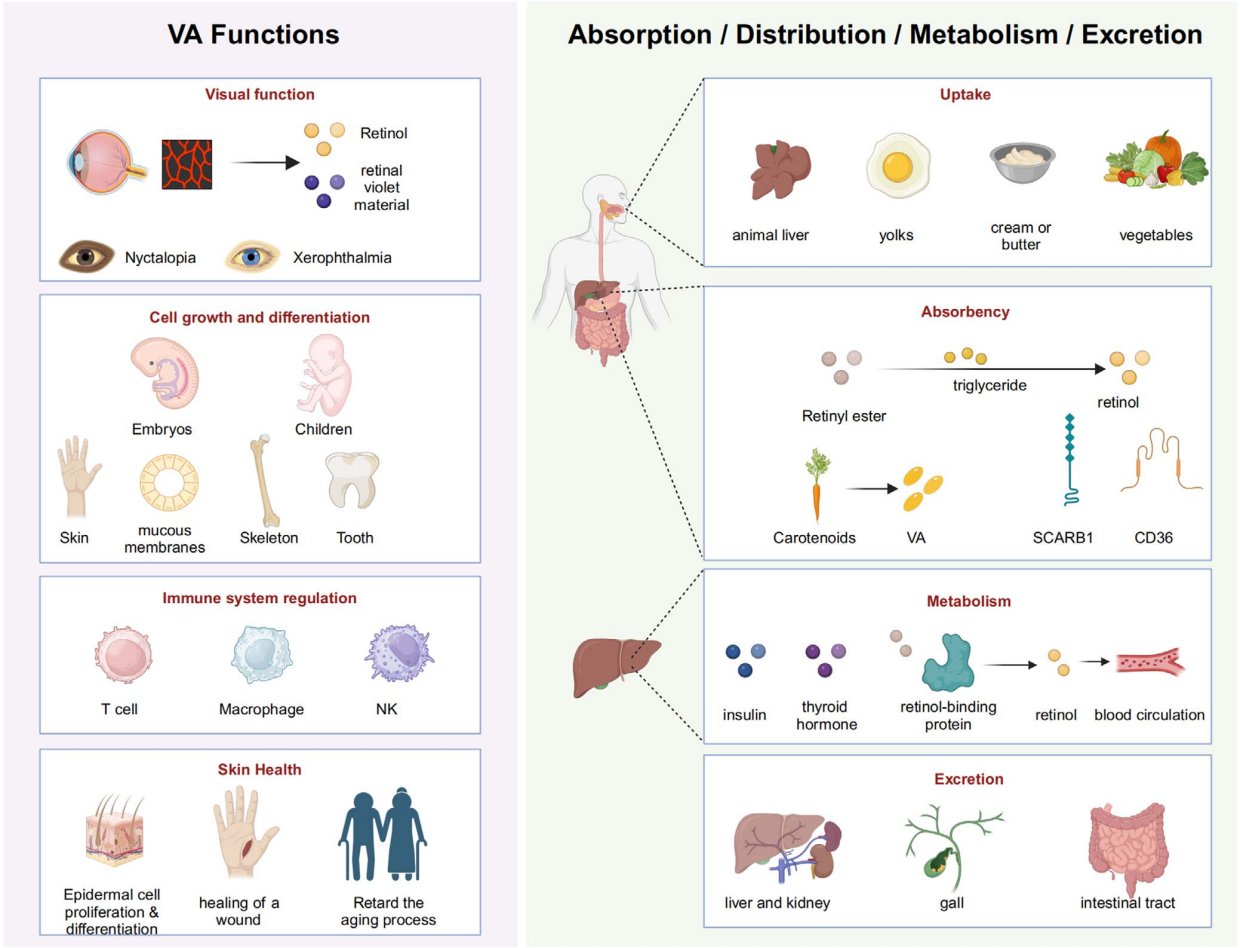
Functions and metabolism of vitamin A. Vitamin A (retinol) is involved in the synthesis of rhodopsin in the visual system. Its deficiency can lead to night blindness and dry eye syndrome. It also regulates cell growth and differentiation (such as in the epidermis and bones) and participates in immune regulation by influencing T cells, macrophages, and other immune cells. Its metabolism depends on retinyl esters in animal livers, egg yolks, and other dietary sources, as well as carotenoids in vegetables. It is absorbed through the intestines and stored in the liver. Excess amounts can be excreted through the liver and kidneys.

Vitamin A is a vital micronutrient essential for various physiological functions in the human body (Figure 1), with its effects encompassing:

1. Visual function maintenance: Retinol, a form of vitamin A, is a key component of visual pigments, particularly rhodopsin, in the retina (Tian et al. 2021). Upon light exposure, rhodopsin binds with retinol, enabling the transmission of visual signals (Xiao 2002). Vitamin A deficiency can result in night blindness, characterized by impaired vision in low‐light conditions, and may also lead to dry eyes, or in severe cases, total blindness (Chinese Society of Preventive Medicine, C.H.B 2024; Dewett et al. 2021; Thirunavukarasu et al. 2022).
2. Cell growth and differentiation: Vitamin A plays a significant role in regulating cell growth, apoptosis, and differentiation, processes vital for embryonic development and childhood growth (Sajovic et al. 2022). It supports the normal growth and repair of skin, mucosal tissues, bones, and teeth, promotes bone development, and is indispensable for maintaining immune system function (Sajovic et al. 2022).
3. Immune system regulation: Vitamin A is integral to immune function, significantly modulating the proliferation and differentiation of T lymphocytes (Abdelkader et al. 2022; Mora et al. 2008). It also enhances the activity of macrophages and natural killer cells, improving the body's ability to combat pathogens and foreign substances (Sirisinha 2015). Additionally, Vitamin A regulates the inflammatory response, playing a key role in maintaining mucosal immunity (Sirisinha 2015).
4. Skin health promotion: Vitamin A is essential for skin health (VanBuren and Everts 2022). It promotes the proliferation and differentiation of epidermal cells, contributing to skin integrity and facilitating wound healing (Wang et al. 2019). Moreover, vitamin A is beneficial in the treatment of skin conditions such as acne and eczema and may help slow skin aging by reducing the appearance of wrinkles and pigmentation (Khalil et al. 2017; Wang et al. 2023; Kafi et al. 2007; Li et al. 2013).

## 2 Eye Diseases

Vitamin A is crucial for maintaining eye health and visual function, primarily by promoting dark adaptation (night vision) and preserving the integrity of the corneal epithelium (Xiao 2002). Its deficiency can lead to damage to the corneal epithelial cell structure and function, causing dry eyes (conjunctival/corneal drying), bitot spot, corneal softening, night blindness, and even blindness (Lee and Jee 2025; McLaughlin et al. 2014). Night blindness is often the first ocular symptom of vitamin A deficiency and can be complicated by corneal infection and perforation in severe cases (Lee et al. 2015). Vitamin A deficiency is a common cause of corneal ulcers, particularly in individuals with malnutrition, malabsorption, or intestinal diseases (Lee et al. 2015; Colburn et al. 2009). Therefore, vitamin A deficiency should be evaluated and corrected in patients with corneal ulcers, and nutritional management should be strengthened in high‐risk groups to prevent vitamin A deficiency.

RA, an active metabolite of vitamin A, regulates axial growth through multiple mechanisms in form‐deprivation myopia (FDM). It is mainly synthesized by retinal pigment epithelial cells (RPE), and its classic active form is ATRA, which can combine with nuclear receptors retinol receptor (RAR) and retinoic acid X receptor (RXR) to form heterodimers and regulate the expression of target genes (Feng et al. 2023). For example, the levels of RA and the expression of retinal dehydrogenase 2 are increased in FDM animal models, accompanied by upregulation of RA binding proteins CRABP‐I and RAR‐β (Huang et al. 2011). Form deprivation inhibits the expression of retinol dehydrogenase 5 (RDH5) through negative feedback, thereby maintaining RA homeostasis (Mao et al. 2021). In the signaling pathway, ATRA stimulates RPE cells to secrete transforming growth factor‐β2 (TGF‐β2) through the phospholipase C (PLC) pathway, but not the adenylyl cyclase pathway. PLC inhibitors can block this process and promote the development of myopia (Zhang et al. 2019). In addition, the changes of RA signaling in the retina–chorio–sclera in the FDM model suggest that RA may participate in the retina–sclera signaling cascade and promote axial elongation by regulating scleral extracellular matrix remodeling (such as glycosaminoglycan synthesis) (Brown et al. 2022). However, the specific signal transduction mechanism still requires further study.

A survey study found no significant association between adolescent dietary vitamin A intake and myopia development in early adulthood; however, the protective effects of Vitamin A‐rich foods during childhood and adolescence remain uncertain (Ng et al. 2020). Emerging studies have shown that RA and its receptors play a key role in eye development and experimental myopia (Ding et al. 2010). Several studies have identified significantly lower serum vitamin A levels in preschool children with abnormal visual development, considering this an independent risk factor for visual impairments in this age group (Wang and Huang 2020; Zhang 2019). Additionally, high doses of vitamin A and vitamin B have been shown to reduce the prevalence of glaucoma (Han and Fu 2022). Early intramuscular injection of high‐dose vitamin A can improve retinal function in infants at high risk of retinopathy of prematurity (ROP) at 36 weeks of gestation (Mactier et al. 2012). However, there was no significant effect on the overall course of ROP (Comander et al. 2023). Future trials should focus on confirming the efficacy of oral vitamin A in reducing the incidence and severity of ROP in preterm infants.

In summary, vitamin A is essential for maintaining normal visual function and preventing eye diseases. As one of the leading causes of vision loss worldwide, interventions targeting visual impairment associated with vitamin A deficiency, especially among children from low socioeconomic backgrounds, need to be strengthened urgently (Xu et al. 2021). Table 1 summarizes 11 studies exploring the relationship between vitamin A and eye diseases.

**TABLE 1:** Studies on the relationship between vitamin A and eye diseases.

Study type	Author	Year	Country	Sample size	Age	Main findings
Systematic review and meta‐analysis	Han and Fu (2022)	2022	Multiple countries	262,189	> 40 years	High doses of vitamin A and vitamin B intake were associated with a lower prevalence of glaucoma
	Batais et al. (2024)	2024	Multiple countries	1101	Gestational age < 32 weeks	Vitamin A and propranolol have not shown efficacy in preventing ROP
	Xu et al. (2024)	2024	Europe	Not explicitly stated	Not explicitly stated	Although vitamin A supplementation may not have an independent effect on myopia, vitamin A ‐related intraocular processes (such as by affecting retinoic acid levels) may indirectly affect the development of myopia. The specific mechanisms include changes in extracellular matrix metabolism, retinal level involvement, and possible compensatory protective mechanisms
Retrospective study	Comander et al. (2023)	2023	America	799	Not explicitly stated	For the overall cohort of RP (retinitis pigmentosa) patients, vitamin A supplementation had no significant beneficial effect on disease progression
Prospective clinical trials and retrospective controlled studies	Garofoli et al. (2020)	2020	Italy	62	Gestational age < 32 weeks	Oral vitamin A supplementation may have a positive effect on reducing the risk of severe ROP in VLBW preterm infants
Case series study	McLaughlin et al. (2014)	2014	Glasgow, United Kingdom	3	They were 34, 41, and 49 years old, respectively	Vitamin A supplementation can significantly improve visual outcomes in patients with xerophthalmia caused by vitamin A deficiency
Prospective cohort study	Ng et al. (2020)	2020	Australia	642	18–23 years	Adequate vitamin A intake was associated with A lower risk of myopia, but after adjusting for potential confounders (such as eye sunlight exposure, education level, and parental myopia), this association was no longer significant, that is, total vitamin A intake in adolescence was not significantly associated with refractive error at age 20 years
Case–control study	Ding et al. (2010)	2010	China	276	7–68 years	No association was found between retinoic acid (RA) and myopia in this study
	Wang and Huang (2020)	2020	China	71	4–14 years	The levels of serum zinc, iron, copper, selenium and vitamin A in most children with strabismus and amblyopia are decreased, and the abnormal rate may be related to the level of serum zinc, iron, copper, selenium and vitamin A
	Zhang (2019)	2019	China	600	< 6 years	The serum vitamin A level of preschool children with visual dysplasia is significantly lower than that of healthy children. Low serum vitamin A level is an independent risk factor for visual impairment in preschool children
Prospective, multicenter, randomized controlled trial	Sun et al. (2020)	2020	China	262	Less than 28 weeks of gestation and < 96 h after birth	Vitamin A supplementation is associated with reduced rates of type 1 retinopathy of prematurity (ROP)

## 3 Vitamin A and Respiratory Diseases

### 3.1 The Significance of Vitamin A in the Development of the Lung

As a fat‐soluble vitamin, vitamin A plays a key role in lung development, differentiation, and the maintenance of function through multiple pathways (Liu 2023). It can regulate alveolar formation in early life and participate in the maintenance and regeneration of lung tissue (Hind et al. 2009). At the same time, it is essential to maintain the elastic retracting force in the lung (Massaro and Massaro 2010). RA, as the active form of vitamin A, is the core regulator of respiratory system development (Timoneda et al. 2018). Ra regulates gene expression by binding and activating two types of nuclear receptors, RAR and RXR, and plays an important role in alveolar formation, lung repair, and remodeling (Timoneda et al. 2018). Fernandes‐Silva et al. (2020) demonstrated that RA regulates lung development through mediating cell signaling pathways, and it enhances the expression of surfactant protein B (SP‐B) through RA receptors in lung tissue, which is a key factor in promoting phospholipid synthesis (Marquez and Chen 2020). In addition, RA signaling is involved in the development of the trachea, airway, lung, and diaphragm during the embryonic period. It continues to affect the health of the respiratory system after birth (Marquez and Chen 2020).

### 3.2 Role of Vitamin A in Respiratory Tract Infections

Respiratory tract infections caused by pathogenic microorganisms are a significant global health issue, particularly in children. Vitamin A maintains respiratory health through two major mechanisms (Mora et al. 2008). First, the respiratory mucosa serves as the primary line of defense against pathogens, and its integrity relies on vitamin A support. Vitamin A can promote the differentiation and repair of mucosal epithelial cells, thereby strengthening the barrier function of the infection defense field (Mora et al. 2008). Secondly, vitamin A is a key regulatory factor of immune cell function, playing a crucial role in the activation and differentiation of T cells and B cells; it can regulate the humoral and cellular immunity of the body, enhance the immune response mediated by Th2 cells, promote the expression of Th1 cells to Th2 cells, and improve the anti‐infection ability of the respiratory tract of the body (Chen 2023). In addition, vitamin A can regulate inflammatory responses and balance immune activities; its derivative, RA A, can inhibit the effect of TNFα on lung epithelial cells and prevent tissue damage caused by excessive inflammation (Zhang, Wang, and Wang 2023).

### 3.3 Vitamin A and Pneumonia

Vitamin A supplementation is generally believed to reduce the risk of acute respiratory infections (ARTIs) (Marquez and Chen 2020; Diness et al. 2011). The lower the vitamin A level in umbilical cord blood, the more likely the infant is to have a neonatal respiratory tract infection during the neonatal period (Wei et al. 2024). However, some studies have shown no significant impact of vitamin A supplementation on the incidence of acute respiratory diseases. In cases where vitamin A supplementation exceeds the recommended dose by the World Health Organization (WHO), there may be an increased incidence of ARTI, especially in children with normal nutritional status (Zhang et al. 2021; Relan et al. 2023). Vitamin A deficiency is a known risk factor for neonatal pneumonia, and a recent systematic review and meta‐analysis indicated that vitamin A supplementation can improve the severity of neonatal pneumonia and reduce mortality (Li et al. 2024). Preventing vitamin A deficiency in neonates and ensuring adequate vitamin supplementation are crucial for improving neonatal health outcomes. This is because vitamin A can enhance the integrity of respiratory epithelial cells and immune defense functions. When the level of vitamin A in the body is insufficient, the respiratory mucosa is vulnerable to pathogen invasion, and its repair ability declines after infection (Quiles et al. 2024; Gong 2025). When there is a deficiency of vitamin A, the ciliary epithelial cells in the respiratory tract are replaced by squamous epithelium, mucus secretion decreases, the basement membrane of the alveoli thickens, collagen ectopic deposits occur, and the structure of the cell matrix changes, which leads to the risk of pathogen invasion (Quiles et al. 2024; Gong 2025).

Several studies have reported a decrease in vitamin A levels in patients with COVID‐19, particularly in severe cases (Tepasse et al. 2021; Tomasa‐Irriguible et al. 2021; Sarohan et al. 2022; Al‐Saleh et al. 2022). Li et al. (2020) further explored this finding, demonstrating an antiviral effect of vitamin A against SARS‐CoV‐2; its antiviral mechanism has the characteristics of multi‐target and multi‐pathway synergy, such as: in the trans‐endothelial migration pathway of white blood cells, by regulating core targets such as ICAM1, MAPK14, IL10, and EGFR, it promotes cell proliferation and repair, maintains the integrity of the respiratory epithelial barrier, reduces the chance of viral invasion, inhibits excessive inflammatory signals caused by viral infection, reduces the migration of inflammatory cells to the lung, and alleviates the immunopathological damage of the lung (Li et al. 2020). Promote antibody production in the B‐cell receptor signaling pathway, forms an immune‐inflammatory regulatory network, and jointly combats SARS‐CoV‐2 infection. It enhances the antioxidant capacity and repair function of host cells in signaling pathways such as FoxO and VEGF, maintains cellular homeostasis, and reduces susceptibility to viral infection (Li et al. 2020). It can also function through antioxidant enzymes such as catalase (CAT). CAT can eliminate reactive oxygen species (ROS) produced during viral infection, reduce oxidative stress damage to lung cells, and improve lung function, etc. (Li et al. 2020). Additional research found that vitamin A levels were significantly negatively correlated with the prognosis of COVID‐19 (Yilmaz et al. 2023). However, a study showed no significant benefit of vitamin A supplementation compared to usual care on outcome severity in hospitalized COVID‐19 patients (Somi et al. 2024). On a more positive note, patients experiencing olfactory dysfunction after coronavirus infection exhibited significant clinical improvement when using 10,000 IU of topical vitamin A combined with olfactory training (OT), compared to OT alone (Helman et al. 2022).

Zhu et al. (2020) and Meng et al. (2021) identified a significant association between vitamin A levels and the severity of community‐acquired pneumonia (CAP), suggesting that vitamin A could serve as a potential biomarker for monitoring disease progression. Low serum vitamin A levels were linked to an increased risk of extrapulmonary complications. In pneumonia treatment, vitamin A supplementation has been considered a beneficial adjunctive therapy, aiding in the reduction of hospital stay duration and pulmonary effusion persistence (Sharma et al. 2024; Li et al. 2022; Cao et al. 2024). However, (Ni et al. 2005) reported contrasting findings, indicating that adjuvant vitamin A therapy did not significantly impact mortality, morbidity, or clinical outcomes in children with non‐measles pneumonia. This difference suggests that the therapeutic effect of vitamin A supplementation may be pathogen‐specific and related to the vitamin A deficiency level and the dose of supplementation in the subjects. Further research is needed to determine its effectiveness in treating pneumonia caused by various pathogens (Chen et al. 2020).

### 3.4 Vitamin A and Recurrent Respiratory Tract Infections (RRTIs)

RRTIs are a common issue in children, significantly affecting health, growth, and development due to their chronic and recurrent nature. Tian et al. (2021) observed a high prevalence of vitamin A deficiency in children with RRTI, with a significant negative correlation between vitamin A levels and RRTI incidence, suggesting that monitoring serum vitamin A levels could be useful for evaluating disease severity. This conclusion has been confirmed by several studies: vitamin A deficiency is closely related to the severity of respiratory tract infection symptoms in children (Zhang, Dai, et al. 2023; Abdelkader et al. 2022). Moreover, research by Zhu et al. (2019) demonstrated that combining vitamin A and vitamin E with sodium carboxymethyl starch significantly improved oxidative stress and immune function in children with RRTI, resulting in better clinical outcomes. The mechanism of action is that retinol and RA can enhance the anti‐inflammatory and bactericidal ability of respiratory immune cells by stimulating neutrophils to produce superoxide, improve the phagocytic efficiency of phagocytic cells to pathogens, and thus reduce the burden of pathogenic bacteria and inflammatory response in vivo (Shi et al. 2023). Therefore, by monitoring the level of vitamin A and supplementing it in a timely manner, the respiratory immune barrier can be enhanced and the risk of infection recurrence can be reduced. Table 2 summarizes 26 studies investigating the relationship between vitamin A and respiratory infections.

**TABLE 2:** Studies on the relationship between vitamin A and respiratory diseases.

Study type	Author	Year	Country	Sample size	Age	Main findings
Systematic review and meta‐analysis	Zhang et al. (2021)	2021	Multiple countries	50,944	0–11 years	Vitamin A supplementation (VAS) has no significant effect on the incidence and course of acute respiratory infection (ARTI). Even more than the recommended dose of VAS by WHO can increase the incidence of ARTI, especially in children with normal nutritional status
	Relan et al. (2023)	2023	Multiple countries	no specific sample size was mentioned	29 days −19 years	The benefit of early adjuvant therapy with vitamins A and D and zinc on clinical outcomes of pneumonia and bronchiolitis is less convincing
	Li et al. (2024)	2024	Multiple countries	21,940	Not explicitly stated	Vitamin A deficiency is a risk factor for neonatal respiratory diseases, and vitamin A supplementation is an effective treatment for neonatal respiratory diseases. Despite the presence of publication bias, sensitivity analysis showed the stability and reliability of meta‐analysis results
	Cao et al. (2024)	2024	Not specified	1485	1 month −14 years	Vitamin A supplementation in the treatment of children with *mycoplasma pneumoniae* pneumonia can significantly improve the clinical efficacy, shorten the length of hospital stay, the disappearance time of pulmonary rales, the duration of cough and fever, reduce the levels of IL‐6 and IL‐10, reduce the inflammatory response, and does not increase the incidence of adverse reactions
	Sharma et al. (2024)	2024	Multiple countries	470	> 18 years	Some antioxidants, such as vitamin A and C, and zinc, may be beneficial in reducing complications from SARS‐CoV‐2, especially when used in combination
	Li et al. (2022)	2022	Multiple countries	3496	Not explicitly stated	Vitamin A combined with conventional therapy can improve the clinical efficacy of children with pneumonia, effectively enhance fever, cough, lung rales, choking milk, chest X‐ray, length of hospital stay, perioral cyanosis and other symptoms, but has no significant effect on mortality
	Helman et al. (2022)	2022	Multiple countries	1325	The mean age was 55 years	Patients with olfactory dysfunction had significant clinical improvement with 10,000 IU of topical vitamin A plus olfactory training compared to olfactory training alone
	Ni et al. (2005)	2005	Tanzania, Brazil, Ecuador, China	1740	1 month −14 years	As an adjuvant treatment for non‐measles pneumonia in children, vitamin A did not significantly reduce mortality or impact the length of hospital stay. High‐dose vitamin A supplementation was associated with increased disease severity, whereas low‐dose vitamin A significantly reduced the recurrence rate of bronchopneumonia
Case–control study	Sarohan et al. (2022)	2022	Turkey	50	> 16 years	Serum retinol levels in patients with severe COVID‐19 were significantly lower than those without COVID‐19 symptoms and were independent of age, sex, and comorbidities
	Li et al. (2021)	2021	China	194	Not explicitly stated	Lower vitamin A levels are closely associated with an increased risk of extrapulmonary complications in children with MPP
	Tian et al. (2021)	2021	China	270	0–14 years	Vitamin A levels are negatively correlated with the incidence of recurrent respiratory tract infections (RRTIs)
	Zhang, Dai, et al. (2023)	2023	China	2592	6 months‐ 14 years	Insufficient or deficient serum vitamin A levels are positively correlated with the occurrence of recurrent respiratory tract infections (RRTIs)
Randomized controlled Trial	Saied et al. (2022)	2022	Cairo	75	1–60 months	Dose: < 1 year: d1, d5,50,000 IU/day；1–5 year: d1, d5,100,000 IU/day Drug‐delivery way: per os Duration of treatment:5 days Conclusion: Zinc or vitamin A supplementation as an adjuvant treatment for community‐acquired pneumonia is beneficial in reducing both the length of hospital stay and the duration of fluid accumulation in children under 5 years of age with pneumonia
	Jiang et al. (2023)	2023	China	92	2–11 years	Dose: 1500 IU/d Drug‐delivery way: per os Duration of treatment: 2 weeks Conclusion: The combination of vitamin A and vitamin D drops and glucocorticoid inhalation for treating Mycoplasma pneumonia in children can promote faster symptom resolution and reduce clinical signs
	Liang et al. (2024)	2024	China	78	2–6 years	Dose: 50000 IU/d Drug‐delivery way: per os Duration of treatment:1 month Conclusion: vitamin A can improve immune function and promote the resolution of symptoms

	Chen et al. (2020)	2020	China	133	1 month‐5 years	Dose: 1 mg/qn Drug‐delivery way: per os Duration of treatment: 60 days Conclusion: vitamin A levels are reduced in children with severe pneumonia caused by various pathogenic infections, with the lowest levels observed in children with *Mycoplasma pneumoniae* pneumonia
	Zhu et al. (2019)	2019	China	100	2–14 years	Dose: 1 mg/d Drug‐delivery way: per os Duration of treatment: 6 month Conclusion: Combined treatment with vitamin A, vitamin E, and sodium carboxymethyl starch can improve oxidative stress and immune function in children with recurrent respiratory tract infections, thereby enhancing clinical efficacy
	Kartasurya et al. (2012)	2012	Indonesia	826	2–5 years	Dose: 60 mg/d Drug‐delivery way: per os Duration of treatment: 1 days Conclusion: vitamin A status modifies the efficacy of Zn supplementation in the treatment of upper respiratory tract infections
Zhang et al. (2024)	2024	China	134	5–12 years	Dose: 2000 IU/d Drug‐delivery way: per os Duration of treatment: 15 days Conclusion: vitamin A and vitamin D combined with glucocorticoid and azithromycin are effective in the treatment of severe mycoplasma pneumonia, which can shorten the time of symptom regression
Cross‐sectional study	Tepasse et al. (2021)	2021	Germany	40	Not explicitly stated	Vitamin A plasma levels were significantly reduced during acute inflammation in patients with COVID‐19, with levels notably lower in severe cases compared to moderate cases. Severe reductions in vitamin A levels were strongly associated with an increased risk of ARDS and mortality
	Yilmaz et al. (2023)	2023	Turkey	53	The mean age was 30 years	Vitamin A levels are inversely correlated with indicators of disease severity in patients with COVID‐19

	Abdelkader et al. (2022)	2022	Saudi Arabia	550	2–12 years	The incidence of subclinical vitamin A deficiency and vitamin A deficiency is significantly higher in children with recurrent respiratory tract infections (RRTIs) compared to those with single respiratory tract infections (RTIs) and the control group
	Al‐Saleh et al. (2022)	2022	Saudi Arabia	155	18–95 years	The mean concentration of vitamin A was lower in the COVID‐19 group than in the control group and was associated with worse clinical outcomes
	Tomasa‐Irriguible et al. (2021)	2021	Spain	120	The mean age was 58.7 years	The level of vitamin A in patients with COVID‐19 is low, and the severe cases decline more significantly
Xing et al. (2020)	2020	China	122	0–15 years	Vitamin A deficiency is significantly associated with sMPP, and vitamin A supplementation may help reduce its incidence. Younger children under 6 years of age with sMPP are more likely to experience vitamin A deficiency
Prospective study	Meng et al. (2021)	2021	China	186	1–14 years	Vitamin A levels are inversely associated with the severity of MPP

### 3.5 Vitamin A and Other Lung‐Related Diseases

#### 3.5.1 Vitamin A and Asthma

Asthma is a chronic airway inflammatory disease characterized by airway hyperresponsiveness and reversible airflow obstruction, often accompanied by dyspnea, cough, wheezing, and other symptoms. Epidemiological data show that the number of asthma patients in China exceeds 30 million, of which children account for more than one‐third (Zhang and Hou 2019). The disease has a significant impact on children's physical development and mental health, and places a heavy burden on their growth (Guo and Xu 2020).

Evidence shows that the serum vitamin A level of children with asthma is significantly lower than that of healthy children, and there is a significant correlation between its concentration and the severity of asthma (Marquez and Chen 2020; Bai and Dai 2018; Shi and Li 2022). However, paradoxically, one study found that high maternal vitamin A intake during pregnancy was associated with an increased risk of childhood asthma before the age of 7 years (Hu et al. 2023; Andino et al. 2019). Meanwhile, other studies failed to confirm a significant association between childhood vitamin A intake or serum vitamin A level and asthma susceptibility (Hu et al. 2023). Studies have shown that in asthma, the decrease in RA levels leads to the activation of AP‐1 and PI3K‐AKT–mTOR pathways, driving the expression of proteins such as MMP9 and HCK, promoting the proliferation of ASM cells and collagen synthesis, thereby intensifying airway remodeling and causing airway fibrosis (Defnet et al. 2021). Moreover, it cannot effectively inhibit NF‐κB and AP‐1, resulting in an increase in the secretion of Th2‐type cytokines such as IL‐4 and IL‐13, further aggravating airway inflammation. Therefore, supplementing RA may be helpful for the treatment of asthma.

At the therapeutic level, the improvement effect of vitamin A on lung function in patients with chronic asthma remains controversial. Some studies have reported that supplementing vitamin A (as an antioxidant) has no obvious clinical benefits. However, Liu (2023) confirmed that vitamin A‐assisted therapy can significantly shorten the duration of respiratory symptoms (cough, wheezing) in children with bronchial asthma, improve lung function, reduce inflammatory markers, and increase T cell levels (Gong 2025). Further study revealed that vitamin A may enhance the dexamethasone‐induced growth of asthmatic airway epithelial cells by down‐regulating GILZ expression and activating the MAPK–ERK pathway, thereby improving the efficacy of glucocorticoids in asthma (Niu et al. 2016). In addition, increased serum vitamin A and 25‐hydroxyvitamin D3 (25OHD3) levels are associated with improved lung function and quality of life in children with asthma in remission; monitoring and maintaining appropriate vitamin A levels can help control asthma symptoms and reduce the frequency of attacks (Bai and Dai 2018). Ge Wei's research results suggest that the combined use of vitamin A, vitamin D, budesonide, and montelukast sodium tablets has a significant effect in reducing the degree of inflammatory response and promoting the rapid improvement of clinical symptoms (Ge 2025). This is because vitamin D can regulate the immune function of children, thereby inhibiting the proliferation of airway smooth muscle and airway remodeling; budesonide acts on intracellular glucocorticoid receptors, inhibiting airway inflammatory responses; montelukast sodium inhibits bronchospasm; while vitamin A inhibits Th2 cell differentiation and the production of IL‐4/IL‐13 by converting into RA, activating regulatory T cells (Treg), and promoting the secretion of inhibitory cytokines (such as IL‐10 and TGF‐β), reducing the production of IgE antibodies by B cells, ultimately inhibiting the release of histamine by mast cells, and alleviating allergic reactions (Ge 2025; Lotfi 2024; Yang et al. 2025; Du et al. 2023; Li and He 2022). The combination of the four works through different mechanisms to promote the improvement of clinical symptoms in children. The active metabolite of vitamin A, ATRA, has been proven to induce the expression of ORMDL3 in NIH3T3, BEAS‐2B, and A549 cells in a dose‐nd time‐dependent manner (Zhuang et al. 2013). The mechanism is by activating PKA and promoting the phosphorylation of CREB. It binds to the CRE element in the ORMDL3 promoter region, thereby initiating transcription (Zhuang et al. 2013). However, ORMDL3 is associated with diseases such as asthma, which suggests that the application of vitamin A may have potential risks, and its advantages and disadvantages in the treatment of asthma need to be further studied (Zhuang et al. 2013).

These conflicting or divergent findings, it may be inconsistent with confounding factors (dietary pattern, environmental exposure, genetic background, other nutrient status), characteristics of study subjects (age, race, region, asthma phenotype), life stage (fetal, infant, childhood, adult), disease stage (acute onset, remission, chronic persistence), and the definition of “vitamin A deficiency”. This further highlights the need to further verify the exact role and applicable conditions of vitamin A supplementation in the management of asthma in different populations.

#### 3.5.2 Vitamin A and Respiratory Distress Syndrome (RDS)

Neonatal RDS is characterized by progressive dyspnea shortly after birth, leading to hypoxia, acidosis, and, in severe cases, respiratory failure (Liu et al. 2023). RDS primarily affects preterm infants and contributes significantly to neonatal complications, including bronchopulmonary dysplasia (BPD) and increased mortality (Liu et al. 2023). Animal studies by Tang et al. (2023) revealed that vitamin A deficiency exacerbates neonatal acute respiratory distress syndrome (ARDS) through multiple pathways, including the HIF‐1 signaling pathway and various metabolic routes. Liu et al. (2023) identified cord blood vitamin A levels as significant indicators of RDS occurrence and severity in preterm infants, suggesting that sufficient vitamin A supplementation during pregnancy may reduce both the incidence and severity of RDS. Elfarargy et al. (2022) found that vitamin A deficiency can aggravate the severity of ARDS and can be used as a predictive indicator for the development and severity of ARDS in neonates. Zeng and Gong (2023) found that vitamin A deficiency can regulate the M1‐type polarization of alveolar macrophages and the expression of its markers iNOS and CD86, increase the content of MDA in the lungs, down‐regulate the activity of SOD, up‐regulate the expression of inflammatory factors IL‐6 and TNF‐α and cell apoptosis, and ultimately aggravate the lung injury of ARDS in neonatal rats. Therefore, in neonates, especially premature infants, early vitamin A intervention can improve the severity and prognosis of lung injury in neonatal ARDS (Zeng and Gong 2023).

#### 3.5.3 Vitamin A and Bronchopulmonary Dysplasia (BPD)

BPD, a common respiratory condition in low birth weight preterm infants, is marked by early lung injury and remains one of the most prevalent chronic conditions in this population (Tracy and Berkelhamer 2019). It is associated with high mortality and long‐term health challenges for survivors, affecting their physical and mental development while imposing significant burdens on families and society (González‐Luis et al. 2020).

Despite advances in neonatal care, no definitive treatment for BPD exists, emphasizing the critical need for prevention. Previous research has demonstrated that vitamin A supplementation reduces BPD incidence in preterm infants (Sun et al. 2020; Huang et al. 2021; Ding et al. 2021; Jensen et al. 2020; Naeem et al. 2019). Notably, vitamin A supplementation beginning in the third trimester and continuing postpartum has proven more effective than the standard postpartum‐only approach (Babu and Sharmila 2010). This supplementation was particularly beneficial in preventing BPD or death in low‐risk infants compared to those at higher risk (Rysavy et al. 2021). For the prevention of BPD, monitoring the vitamin A level in the mother's or newborn's body and formulating a vitamin A supplementation plan based on their individual conditions, as well as supplementing vitamin A before or after childbirth, can promote the development of alveoli and pulmonary vessels, reduce the occurrence and severity of the disease, and is of great significance for disease prevention and treatment. Additionally, research by Xie et al. (2018) highlighted the combined benefit of nebulized budesonide and oral vitamin A for ARDS treatment in preterm infants, significantly improving oxygenation, promoting weight gain, reducing the duration of oxygen therapy, and decreasing BPD incidence. These findings underscore the potential of vitamin A as a therapeutic strategy to improve outcomes for at‐risk neonates. Table 3 summarizes 13 studies exploring the relationship between vitamin A and BPD.

**TABLE 3:** Studies on the relationship between vitamin A and bronchopulmonary dysplasia.

Study type	Author	Year	Country	Sample size	Age	Main findings
Systematic review and meta‐analysis	Ding et al. (2021)	2021	Multiple countries	1409	Gestational age < 37 weeks	Vitamin A supplementation helps prevent bronchopulmonary dysplasia in preterm infants
	Huang et al. (2021)	2021	Multiple countries	1499	Gestational age < 37w	Early vitamin A supplementation has a certain preventive and therapeutic effect on bronchopulmonary dysplasia (BPD) in preterm infants, and it is significantly effective in reducing the incidence of BPD on day 28
Randomized controlled Trial	Rysavy et al. (2021)	2021	America	807	Not explicitly stated	Dose: 5000 IU/d Drug‐delivery way: intramuscular Duration of treatment: 12 days Conclusion: The effect of vitamin A therapy on bronchopulmonary dysplasia (BPD) or death was more significant in low‐risk infants than in high‐risk infants
Prospective, multicenter, randomized controlled trial	Sun et al. (2020)	2020	China	262	Gestational age < 28w	Vitamin A supplementation may have a positive effect on reducing bronchopulmonary dysplasia (BPD)
Animal experimental research	Tang et al. (2023)	2023	China	80	Not explicitly stated	Vitamin A deficiency can affect the growth, development and immune function of neonates, and is closely related to the severity of neonatal ARDS
Review	Naeem et al. (2019)	2019	Multiple countries	Not explicitly stated	Gestational age < 37w	In clinical studies, the efficacy of vitamin A supplementation in the treatment of bronchopulmonary dysplasia is also controversial

## 4 Vitamin A and Infectious Diseases

### 4.1 Vitamin A and Human Immunodeficiency Virus (HIV)

HIV is transmitted via blood, sexual contact, or mother‐to‐child transmission, primarily targeting the immune system and, if untreated, often progressing to acquired immunodeficiency syndrome (AIDS). In its early stages, HIV infection may present with few or no symptoms, rendering transmission insidious. As the disease advances, individuals typically experience symptoms such as weight loss, chronic fatigue, fever, and dyspnea, which not only impair quality of life but also heighten the risk of complications. Consequently, preventing HIV infection and providing effective treatment and support remain critical global health priorities.

HIV specifically targets and depletes CD4+ T cells, which are essential for a robust immune response. Research indicates that vitamin A enhances the proliferation and differentiation of CD4+ T cells, thereby strengthening the immune response (Hall, Cannons, et al. 2011). However, individuals with HIV often exhibit deficient vitamin A levels, which may impair immune function and exacerbate symptoms, rendering them more susceptible to infections (Mehta and Fawzi 2007). For instance, a study of HIV‐positive pregnant women and their infants found that maternal vitamin A deficiency significantly increased the risk of mother‐to‐child HIV transmission (Semba et al. 1994). Therefore, supplementing vitamin A in HIV‐positive individuals can not only enhance immune function, delay the progression of the disease, reduce the risk of opportunistic infections, but also improve the pregnancy outcomes of pregnant women. By monitoring the vitamin A level of AIDS patients and supplementing it in time, the quality of life of the patients can be improved. Table 4 summarizes three studies examining the relationship between vitamin A and HIV.

**TABLE 4:** Studies on the relationship between vitamin A and HIV.

Study type	Author	Year	Country	Sample size	Age	Main findings
Animal experiments	Hall, Cannons, et al. (2011)	2011	America	Not explicitly stated	Not explicitly stated	The vitamin A metabolite RA is not only involved in the induction of regulatory T cells, but also plays a key role in inflammatory T cell responses (TH‐1 and TH‐17) by exerting cell‐autonomous effects within T cells through the RARα receptor. It may also indirectly regulate the adaptive immune response by affecting other cell types such as antigen‐presenting cells (apcs)
Review	Mehta and Fawzi (2007)	2007	Not explicitly stated	Not explicitly stated	Not explicitly stated	There is no evidence to recommend vitamin A supplementation for HIV‐Infected pregnant women; However, regular vitamin A supplementation in HIV‐Infected infants and children helps reduce all‐cause mortality and morbidity
Cross‐sectional study	Semba et al. (1994)	1994	Not explicitly stated	338	Not explicitly stated	Maternal vitamin A deficiency can lead to mother‐to‐child transmission of HIV
Randomized controlled Trial	Makinde et al. (2017)	2017	Lagos, Nigeria	50	23–58 years	Dose: 5000 IU/d Drug‐delivery way: per os Duration of treatment: 1 month Conclusion: vitamin A supplementation has no significant regulatory effect and benefit on oxidative stress indicators in HIV patients

### 4.2 Vitamin A and Measles

Measles is a highly contagious viral disease primarily transmitted through respiratory droplets, predominantly affecting children and unvaccinated populations. While universal vaccination has significantly reduced global measles incidence, high morbidity and mortality rates persist in certain developing and impoverished regions. Vitamin A is essential for individuals with measles, as it supports immune function and overall health.

A study by Diness et al. (2011) suggested that vitamin A supplementation at birth may influence susceptibility to measles infection, with gender‐specific variations observed during outbreaks. Despite this, numerous studies provide limited evidence on the efficacy of vitamin A in treating measles in children, highlighting a gap in the literature (Huiming et al. 2005; Huang and Wang 2019; Yao et al. 2017; Zheng et al. 2015). However, available data indicate that vitamin A therapy can help reduce measles‐related mortality in children, particularly when combined with measles vaccination (Huiming et al. 2005; Sudfeld et al. 2010; Mishra et al. 2008; Semba 2003). In contrast, a randomized controlled trial by Bello et al. (2016) failed to demonstrate sufficient evidence that vitamin A can prevent childhood blindness caused by measles, underscoring the need for further high‐quality randomized trials to comprehensively assess vitamin A's role in preventing measles‐related blindness. Table 5 summarizes six studies investigating the relationship between vitamin A and measles.

**TABLE 5:** Studies on the relationship between vitamin A and Measles.

Study type	Author	Year	Country	Sample size	Age	Main findings
Randomized controlled Trial	Huang and Wang (2019)	2019	China	104	6 months–5 years	Dose: <1 year: 100,000 IU >1 year: 200,000 IU Drug‐delivery way: per os Duration of treatment: 5–7 days Conclusion: In the treatment of measles in children, the combination of small doses of IFN‐α and vitamin A can effectively improve the efficacy, improve the symptoms of children, promote rehabilitation, and has good safety
	Yao et al. (2017)	2017	China	98	6 months–12 years	Dose: 25,000 IU Drug‐delivery way: per os Duration of treatment:5 days Conclusion: vitamin A is effective in the treatment of children with measles, which can effectively improve the prognosis of children
	Zheng et al. (2015)	2015	China	100	5 months–13 years	Dose: 25000 IU/d Drug‐delivery way: per os Duration of treatment: 5 days Conclusion: vitamin A in the treatment of children with measles can quickly relieve the clinical symptoms, the curative effect is significant, and there is no adverse reaction
Systematic review and meta‐analysis	Huiming et al. (2005)	2011	Multiple countries	2574	6 months–15 years	Although no significant reduction in overall measles mortality was found in children treated with vitamin A, two doses of vitamin A reduced overall and pneumonia‐specific mortality in children under two years of age
	Sudfeld et al. (2010)	2010	Multiple countries	Not explicitly stated	6 months‐5 years	Measles vaccine and vitamin A treatment are effective interventions to prevent measles deaths in children
	Bello et al. (2016)	2016	Durban and Ndola, Zambia	260	< 18 years	Because the study was not evaluated for the primary outcome, small sample size, possible reporting bias, and missing outcome data, the available evidence is insufficient to prove the effect of vitamin A on preventing blindness in children with measles

## 5 Vitamin A and Metabolic Diseases

### 5.1 Vitamin A and Diabetes

Diabetes mellitus, a metabolic disorder characterized by hyperglycemia, is primarily classified into type 1 and type 2 diabetes. It significantly contributes to various chronic metabolic conditions, including cardiovascular diseases, and is associated with complications such as diabetic nephropathy, retinopathy, and neuropathy (Lotfy et al. 2017). Emerging research highlights the roles of vitamin A and its metabolites in the development and progression of diabetes (Brun et al. 2013). RA, the active form of vitamin A, plays a key role in regulating insulin secretion and supporting pancreatic β‐cell function: it promotes β‐cell proliferation and survival by modulating gene expression, thereby sustaining normal insulin secretion; furthermore, vitamin A is involved in regulating glucose and lipid metabolism, which is essential for preventing insulin resistance, a key pathological factor in type 2 diabetes, often associated with obesity and chronic inflammation (Said et al. 2021; Trasino and Gudas 2015). By reducing inflammation in adipose tissue and enhancing lipid metabolism, vitamin A and its metabolites may reduce the risk of insulin resistance (Liu, Qin, et al. 2023).

Vitamin A is vital for pancreatic cell function in adults, as vitamin A deficiency leads to islet dysfunction through activating of islet cell populations (Zhou et al. 2020). Vitamin A supplementation may help prevent apoptosis and β‐cell loss in diabetic patients while protecting islet β‐cells from autoimmune destruction, reducing islet inflammation, and preserving islet function (Trasino and Gudas 2015; Trasino et al. 2015). Adequate vitamin A intake may help prevent autoimmune diabetes (Trasino and Gudas 2015). The study by Su et al. (2022) suggests that sufficient vitamin A intake may lower diabetes risk, particularly in men. Additionally, circulating vitamin A and vitamin E levels have been shown to correlate with cognitive function in elderly patients with type 2 diabetes, with lower circulating vitamin A levels predicting an increased risk of mild cognitive impairment in these individuals (Huang et al. 2020). Some studies have identified a negative correlation between blood retinol concentrations and type 1 diabetes mellitus (T1DM) and gestational diabetes mellitus (GDM) (Lu et al. 2022). Moreover, Kim et al. (2017) found that serum levels of vitamin A metabolites are linked to the onset of type 2 diabetes (T2DM) and show promise for treating diabetes and its complications (Shirai et al. 2016; Yosaee et al. 2016). Evidence also suggests that high‐dose vitamin A combined with vitamin E and zinc supplementation may improve glycemic control, β‐cell function, and insulin secretion in adults with T2DM, as supported by some animal studies (Said et al. 2021; Trasino and Gudas 2015). Therefore, monitoring and managing vitamin A levels is crucial for improving glucose metabolism and preventing complications of diabetes.

While the potential of vitamins in diabetes management is promising, current research remains limited (Table 6), and clinical data on the quantitative relationship between vitamins and diabetes management are insufficient. As a result, vitamins are not yet recommended as routine treatments for diabetes (Yan and Khalil 2017). Nonetheless, the therapeutic potential of vitamins in diabetes warrants further investigation, and high‐quality studies are needed to clarify their roles in diabetes development and progression, as well as to evaluate their potential applications in diabetes care (Liu, Qin, et al. 2023).

**TABLE 6:** Studies on the relationship between vitamin A and diabetes.

Study type	Author	Year	Country	Sample size	Age	Main findings
Prospective cohort study	Su et al. (2022)	2022	China	17,111	> 18 years	Adequate intake of vitamin A, from both plant and animal sources, may help prevent diabetes, particularly in men
Case–control study	Kim et al. (2017)	2017	Korea	275	Not explicitly stated	Serum levels of vitamin A ‐related metabolites are associated with the incidence of T2DM
Randomized controlled Trial	Said et al. (2021)	2021	Egypt	98	20–60 years	Dose: 50000 IU/d Drug‐delivery way: per os Duration of treatment: 3 month Conclusion: High‐dose supplementation of vitamin A and vitamin E combined with zinc can improve glycemic control, beta cell function, and insulin secretion in adults with T2DM
	Xue (2022)	2022	China	64	5–13 years	Dose: 2000 U/d Drug‐delivery way: per os Duration of treatment: 3 month Conclusion: vitamin A combined with insulin aspart in the treatment of T1DM children can reduce the levels of fasting blood glucose, 2‐h postprandial blood glucose, daily insulin dosage, IL‐17 levels, FGF19 levels and the incidence of complications, and increase the level of serum C‐peptide. The effect is better than that of insulin aspart alone
	Zheng et al. (2024)	2024	China	120	5–13 years	Dose: 2000 U/d Drug‐delivery way: per os Duration of treatment: 3 month Conclusion: vitamin A combined with insulin detemir and insulin aspart in the treatment of children with diabetes mellitus can significantly improve glucose and lipid metabolism, increase blood glucose level, and reduce the incidence of complications
Systematic review and meta‐analysis	Liu, Qin, et al. (2023)	2023	Multiple countries	Not explicitly stated	40–84 years	Some studies have found an inverse association between β‐carotene or vitamin A intake and the risk of diabetes, but some European and American studies have found no significant association. Animal studies have shown that vitamin A deficiency can cause pancreatic β‐cell dysfunction and reduced insulin secretion, and vitamin A can also alleviate inflammation in diabetic patients through antioxidant function, but the overall research results are contradictory
	Lu et al. (2022)	2022	Multiple countries	Not explicitly stated	Not explicitly stated	Serum retinol concentration is negatively correlated with T1DM and GDM
Review	Brun et al. (2013)	2013	Not explicitly stated	Not explicitly stated	Not explicitly stated	Retinoids are necessary for the normal endocrine function of pancreatic β cells. They can regulate insulin secretion and related gene expression, and also participate in the regulation of glucagon secretion by α cells
	Yosaee et al. (2016)	2016	Multiple countries	Not explicitly stated	Not explicitly stated	Vitamin A is involved in the immune regulation and islet function maintenance of type 1 diabetes through a variety of ways
	Trasino and Gudas (2015)	2015	Multiple countries	Not explicitly stated	Not explicitly stated	Vitamin A may be involved in the occurrence and development of diabetes by regulating metabolic pathways, immune function, and the development and function of pancreatic β cells
	Yan and Khalil (2017)	2017	Multiple countries	Not explicitly stated	Not explicitly stated	No clear evidence was found to support a beneficial effect of any particular vitamin in the treatment of T2DM
Animal experimental research	Zhou et al. (2020)	2020	China	Not explicitly stated	Mice aged 6 weeks	Vitamin A maintains islet homeostasis by regulating islet stellate cell function. Vitamin A deficiency can activate islet stellate cells, thereby affecting islet endocrine function
	Trasino et al. (2015)	2015	America	Not explicitly stated	Mice aged 6–7 weeks	Vitamin A plays A key role in maintaining the normal structure of pancreatic islets and the quality and function of endocrine cells in adult mice. Vitamin A deficiency alters endocrine cell characteristics and islet plasticity mainly by affecting islet cell apoptosis and related gene expression, and pancreatic β cells are particularly sensitive to vitamin A deficiency

### 5.2 Vitamin and Growth Hormone Deficiency (GHD)

GHD is an endocrine disorder caused by dysfunction in the anterior pituitary or hypothalamus, and it is a leading cause of short stature in children (Han et al. 2019). Characterized by generalized growth retardation and reduced height, GHD has been linked to altered metabolic and growth processes. Recent studies suggest that vitamin A plays a significant role in the onset and progression of GHD.

Research by Liang et al. (2021) demonstrated that serum vitamin A levels are notably lower in children with GHD and positively correlate with insulin‐like growth factor 1 (IGF‐1) levels. This correlation highlights the importance of monitoring both vitamin A and IGF‐1 levels in affected children, as correcting these deficiencies may lead to more effective treatment and improved outcomes. These findings suggest that vitamin A influences growth and development in children with GHD by modulating IGF‐1 levels, potentially enhancing the efficacy of treatment. Furthermore, investigating the impact of vitamin A deficiency on the effectiveness of recombinant human growth hormone therapy in GHD‐affected children is essential. Understanding this interaction could help in developing personalized treatment strategies, improving overall therapeutic success.

### 5.3 Vitamin and Thyroid Disease

Thyroid disease, a common endocrine disorder, arises from a multifactorial etiology involving genetic, environmental, and nutritional factors. Recent research highlights the crucial role of vitamin A in maintaining thyroid health, with a significant correlation between vitamin A deficiency and thyroid dysfunction. The active forms of vitamin A—retinol and RA—are essential for thyroid hormone synthesis, secretion, and regulation (Capriello et al. 2022).

Zimmermann et al. (2004) found that vitamin A deficiency exacerbates thyroid dysfunction in iodine‐deficient children, whereas vitamin A supplementation improves thyroid function. Therefore, maintaining normal levels of vitamin A is crucial for the stability of thyroid function. This suggests that combined iodine and vitamin A supplementation could be beneficial for preventing and managing thyroid disorders, especially in iodine‐deficient regions. During pregnancy, vitamin A deficiency can lead to anemia and subclinical hypothyroidism, underscoring the importance of monitoring vitamin A levels and providing evidence‐based supplementation during this period (Jia et al. 2022). A study of Arctic residents revealed significantly lower vitamin A levels in women compared to men, with the risk of subclinical hypothyroidism doubling when vitamin A levels fell below 1.39 μmol/L (Elfimova et al. 2023). In conclusion, maintaining normal vitamin A level is of great significance for the stability of thyroid function, and its synergistic effect with iodine nutrition deserves attention in the prevention and control of thyroid diseases.

## 6 Vitamin A and Autoimmune Diseases

### 6.1 Vitamin A and Multiple Sclerosis (MS)

MS is a disease characterized by demyelinating inflammation and neurodegenerative changes in the central nervous system, and it is prevalent among young people. Although the exact etiology and pathophysiology have not been fully clarified, environmental factors are considered to play an important role in the occurrence and progression of the disease, among which vitamin A may be a potential factor.

Studies have shown that vitamin A is involved in the pathological regulation of MS through multiple pathways, including reducing inflammatory responses, promoting autoimmune tolerance, and providing neuroprotective effects (Fragoso et al. 2014). The systematic review by Tryfonos et al. (2019) pointed out that vitamin A and its metabolites can improve the pathological process of MS through mechanisms such as regulating the function of immune cells, enhancing the autoimmune tolerance of the central nervous system, and promoting nerve regeneration. Studies have shown that supplementing with vitamin A in MS patients can significantly regulate the expression of RA receptor (RAR) genes, especially by down‐regulating RAR‐α and up‐regulating RAR‐γ (Bitarafan et al. 2013). These changes suggest that therapeutic effects may be produced through these regulatory pathways. Vitamin A mainly functions in MS by regulating the Th17/Treg cell axis through the active metabolite ATRA: ATRA binds to the nuclear receptor RARα, inhibits the expression of the key transcription factor RORγt in Th17 cells, reduces the secretion of pro‐inflammatory factors such as IL‐17 and IL‐23, and simultaneously induces the expression of the Treg cell‐specific transcription factor FoxP3 through the Smad3 pathway, promoting the release of anti‐inflammatory factors such as IL‐10 and TGF‐β (Elias et al. 2008). In addition, ATRA can inhibit the expression of IL‐6 and IL‐23 receptors to weaken the Th17 differentiation signal; it can also enhance the stability of Treg cells through the IL‐2/STAT5 pathway and bidirectionally regulate the immune balance (Xiao et al. 2008). Clinical evidence further confirms that RA drugs can bring therapeutic benefits to Th1/Th17‐related diseases such as MS through the immune shift of the above‐mentioned Th17/Treg balance (transforming to the anti‐inflammatory phenotype) (Elias et al. 2008; Mohammadzadeh Honarvar et al. 2013; Hall, Grainger, et al. 2011; Schambach et al. 2007).

### 6.2 Vitamin A and Systemic Lupus Erythematosus (SLE)

SLE is a systemic autoimmune disorder marked by an aberrant immune response leading to multi‐organ damage. Although the precise pathogenesis of SLE remains unclear, research indicates that an increased number of Th17 cells is strongly associated with erythema and organ damage in SLE patients (Garrett‐Sinha et al. 2008; Alunno et al. 2012; Shah et al. 2010; Shin et al. 2011).

Vitamin A, through its active form RA, plays a critical role in immune regulation. Numerous studies have highlighted its role in modulating Th17 cell populations and supporting regulatory T (Treg) cell homeostasis (Elias et al. 2008). Research by Handono et al. (2016) demonstrated a correlation between vitamin A levels and the Th17/Treg balance in patients with SLE, underscoring the importance of maintaining this balance to prevent excessive inflammation and preserve immune tolerance. RA's ability to regulate the Th17/Treg ratio suggests its potential to mitigate the immune response in SLE, offering a promising therapeutic avenue for restoring immune equilibrium in affected individuals.

### 6.3 Vitamin A and Ulcerative Colitis (UC)

UC is a recurrent and repulsive inflammatory bowel disease (IBD) with rectal mucosal inflammation as the initial symptom and continuous spread from the distal to the proximal end, and it belongs to an autoimmune disease (Pang et al. 2021). Since the 21st century, UC has become a global disease, with its incidence rate increasing year by year (Alatab et al. 2020). Its pathogenesis involves genetic susceptibility and chronic immune‐mediated intestinal inflammation; among them, intestinal flora disorder and mucosal barrier dysfunction play a key role in the disease progression by driving chronic inflammation (McGuckin et al. 2007; Plichta et al. 2019). The gut microbiota is crucial for the normal development of the immune system and energy metabolism, and its imbalance is closely related to diseases such as IBD (Guarner 2006).

Vitamins A and D, as important fat‐soluble vitamins, by regulating intestinal barrier function (such as regulating the expression of tight junction proteins ZO‐1, Occludin, and Claudin) and mucosal immune responses (such as inducing ILC3 to produce IL‐22, inhibiting the secretion of IFN‐γ and IL‐17 by Th1/Th17 cells, and inducing regulatory T cells maintain intestinal homeostasis and indirectly affect the composition of the intestinal microbiota (Cantorna et al. 2019)). Their deficiency can lead to a decrease in microbiota diversity, disrupt the balance of the intestinal microbiota, weaken mucosal barrier function (such as reduced expression of tight junction proteins), increase mucosal permeability, increase the secretion of pro‐inflammatory cytokines (such as IL‐12, IFN‐γ), damage the integrity of the barrier, and increase the risk of intestinal infection and inflammation (Amimo et al. 2022; Xiao et al. 2018; Zhou et al. 2021). It is suggested that both are crucial for maintaining intestinal homeostasis.

In the high‐sugar and high‐fat diet (HFSD) mouse model, vitamin A supplementation (VAS) can maintain the α diversity of the intestinal flora, prevent the reduction of *Porphyromonadaceae* and the increase of RC9 genus caused by HFSD, enrich the beneficial bacteria *Lachnospiraceae*, and may exert a protective effect through the gut‐brain axis (Biyong et al. 2021). Study on dextran sulfate sodium (DSS) mice with different doses of ATRA showed that both 15 and 30 mg/kg ATRA could reduce the disease activity index (mDAI) and colonic histological score, increase colonic length, and lower the levels of TNF‐α in serum and colonic tissue, the effect was more significant in the high‐dose group (Feng et al. 2016). However, there are limitations in the repair of intestinal mucosal structure. Relevant animal experiments have found that the above therapeutic effects are achieved by regulating the intestinal flora, vitamin A can enhance the diversity of the flora, increase the abundance of short‐chain fatty acid (SCFAs) producing bacteria such as *Akkermansia* and *Lactobacillus*, and raise the levels of acetic acid and butyric acid in the cecum, furthermore, it repairs intestinal barrier proteins (Muc4, ZO‐1) and inhibits inflammatory factors such as TNF‐α and IL‐6, the fecal microbiota transplantation experiment confirmed that its therapeutic effect depends on the regulation of the microbiota (Pang et al. 2021). Zhang et al. (2016) found that the combined application of 1,25‐(OH)₂D₃ and ATRA could significantly improve the symptoms of UC in mice, reduce colonic tissue damage, lower the disease activity index (DAI) and myeloperoxidase (MPO) activities, and increase colonic length. Meanwhile, the expression of Treg cell Foxp3 and its related cytokines TGF‐β and IL‐10 in colonic tissue was up‐regulated, and the effect was superior to that of monotherapy; moreover, ATRA could antagonize the increase of blood calcium induced by 1,25‐(OH)₂D₃ and improve renal function (Zhang et al. 2016).

Cell experiment showed that vitamin A could improve LPS‐induced inflammatory intestinal barrier dysfunction and enhance the expressions of ZO‐1, Occludin, and Claudin‐1 in the tight junction region of LPS‐induced iepc‐j2 cells (He et al. 2019). 0.1 μmol/L vitamin A can significantly enhance the inflammatory intestinal barrier function induced by LPS. Double‐blind, randomized controlled trials have shown that oral vitamin A can improve the clinical remission rate, symptom manifestations, and mucosal healing rate of patients with UC; however, larger sample sizes and long‐term follow‐up are needed for verification (Masnadi Shirazi et al. 2018). To summarize, vitamin A plays multiple roles in the prevention and treatment of UC by regulating the intestinal flora‐barrier‐immune axis.

## 7 Toxicity of Vitamin A

Vitamin A poisoning is relatively rare and is usually caused by excessive intake of dietary supplements, liver, eggs, and other fortified foods, or treatment with vitamin A‐like drugs (McLaughlin et al. 2014). When the plasma retinol level exceeds 2.09 μM, it can be diagnosed as hypervitaminosis A (Carazo et al. 2021). There are differences in the types of poisoning and dose characteristics. The trigger dose for acute poisoning is > 500 mg/day for adults and > 30 mg/day for infants. The threshold for those with renal failure or alcoholism will decrease (Jing et al. 2016; Clugston and Blaner 2012). Chronic poisoning is more than 10 mg/day for several months in adults, and the trigger dose for children is 7.5–15 mg/day (Carazo et al. 2021). The clinical manifestations are specific to the target organs. In the nervous system, pseudo‐brain tumors related to intracranial hypertension may occur, presenting as headache, optic disc edema, and consciousness disorders (Lagacé et al. 2024). The digestive system can cause liver damage due to elevated triglyceride levels, which activate hepatic stellate cells and trigger fibrosis or even liver cirrhosis, accompanied by elevated liver enzymes and portal hypertension (Carazo et al. 2021). The skeletal system suffers from hypercalcemia due to excessive vitamin A intake. Excessive vitamin A may enhance the activity of osteoclasts and promote the release of bone calcium into the bloodstream, leading to bone pain, osteoporosis, osteomalacia, and pathological fractures (Borgan et al. 2022; Lorenzo et al. 2020). In terms of skin and mucous membranes, local medication can cause erythema and desquamation, while systemic exposure can lead to dry skin and shedding of mucous membranes (Carazo et al. 2021). It has been found in the reproductive system that long‐term chronic excessive intake of vitamin A can interfere with the metabolism of RA and the expression of genes related to spermatogenesis, leading to abnormal sperm morphology and impaired function in mice, suggesting that excessive intake of vitamin A in humans may pose male reproductive risks (Yokota et al. 2021). Even exposure in the early stage of pregnancy poses teratogenic risks, including abnormalities in the fetus's craniofacial, heart, thymus, and central nervous system (Carazo et al. 2021). In addition, there are special toxic reactions, such as RA syndrome related to isotretinoin treatment, which is manifested as dyspnea, pleural effusion, and multiple organ failure (Patatanian and Thompson 2008). During treatment, the source of vitamin A should be immediately discontinued. Most acute symptoms can be reversed after drug withdrawal. At the same time, symptomatic treatment can be provided, such as using acetazolamide to lower intracranial pressure (Carazo et al. 2021). For long‐term medication, regular monitoring of liver enzymes, bone density, and blood lipids is required (Carazo et al. 2021). It is worth noting that carotenoids (such as beta‐carotene, etc.) are relatively safe and only cause reversible skin jaundice (Carazo et al. 2021). However, smokers need to be cautious. A Finnish cohort study has shown that high intake of beta‐carotene may increase the risk of lung cancer (Blumberg and Block 1994). Therefore, excessive intake of foods and supplements rich in vitamin A should be avoided, especially for pregnant women, children, and those with impaired liver or kidney function, who need to strictly control their intake. For individuals on a plant‐based diet, it is essential to assess their total intake of vitamin A.

## 8 Prospects and Outlook

Vitamin A plays a critical role in immune regulation, cell differentiation, and the maintenance of epithelial integrity, underscoring its importance in preventing and managing a range of clinical conditions. Research investigating the relationship between vitamin A and various diseases—including infectious diseases, metabolic disorders, autoimmune diseases, nutritional eye conditions, and pediatric growth—has garnered significant interest. In autoimmune disorders such as SLE and MS, vitamin A has been shown to modulate T cell subset balance, suggesting its potential as an adjunctive therapeutic agent. Ophthalmological studies further highlight that vitamin A deficiency can led to conditions like corneal ulcers and myopia, emphasizing its critical role in pediatric eye health. However, challenges persist. While associations between vitamin A and various diseases are recognized, the specific mechanisms, especially across different age groups and disease stages, remain inadequately understood. Moreover, research has predominantly focused on vitamin A deficiency, with limited exploration of the health implications of excess vitamin A and the dose–response relationship. Future research should explore how maintaining the optimal vitamin A level affects disease progression and treatment outcomes. Key areas for future research include: (1) developing standardized and systematic experimental designs to accurately assess vitamin A's role in diverse diseases; (2) Carry out a large‐scale longitudinal study. This study can adopt a multicenter longitudinal design, systematically control confounding factors such as dietary patterns, stratify the cohort by age, race, and asthma phenotype, and combine disease staging to explore the relationship between vitamin A intake, health indicators, and disease progression, especially for the situation of children; (3) investigating vitamin A's interactions with other nutrients to better understand its broader public health applications. In conclusion, vitamin A holds considerable promise in clinical disease management, but a deeper understanding of its mechanisms and benefits across conditions is essential for optimizing interventions and improving health outcomes in children and high‐risk populations.

## Author Contributions

**Shuhan Rao:** conceptualization (lead), data curation (lead), formal analysis (lead), methodology (lead), software (lead), writing – original draft (lead). **Tiewei Li:** conceptualization (equal), data curation (equal), formal analysis (equal), methodology (equal), software (equal), writing – original draft (equal). **Xiaojuan Li:** data curation (supporting), funding acquisition (lead), resources (equal), validation (equal). **Ligong Hou:** project administration (lead), supervision (lead), visualization (equal), writing – review and editing (equal). **Wan Sun:** data curation (supporting), resources (supporting), validation (supporting).

## Disclosure

The authors have nothing to report.

## Ethics Statement

This review did not require ethical approval as it was based on previously published studies.

## Data Availability

The authors have nothing to report.
